# How environment geometry affects grid cell symmetry and what we can learn from it

**DOI:** 10.1098/rstb.2013.0188

**Published:** 2014-02-05

**Authors:** Julija Krupic, Marius Bauza, Stephen Burton, Colin Lever, John O'Keefe

**Affiliations:** 1 Department of Cell and Developmental Biology, University College London, London WC1E 6BT, UK; 2 Sainsbury Wellcome Centre, University College London, London WC1E 6BT, UK; 3 Institute of Behaviour Neuroscience, University College London, London WC1H 0AP, UK; 4 Department of Psychology, Durham University, Durham DH1 3LE, UK

**Keywords:** grid cell, spatially periodic cells, boundary cell, border cell, hippocampus, symmetry

## Abstract

The mammalian hippocampal formation provides neuronal representations of environmental location but the underlying mechanisms are unclear. The majority of cells in medial entorhinal cortex and parasubiculum show spatially periodic firing patterns. Grid cells exhibit hexagonal symmetry and form an important subset of this more general class. Occasional changes between hexagonal and non-hexagonal firing patterns imply a common underlying mechanism. Importantly, the symmetrical properties are strongly affected by the geometry of the environment. Here, we introduce a field–boundary interaction model where we demonstrate that the grid cell pattern can be formed from competing place-like and boundary inputs. We show that the modelling results can accurately capture our current experimental observations.

## 1. Introduction

Grid cells represent a cell class in medial entorhinal cortex (mEC) which is active in multiple fields spanning the entire environment and arranged in a grid of equilateral triangles exhibiting hexagonal symmetry [1]. Initial experiments suggested that they preserve their symmetry across different familiar environments changing only the offsets of their fields relative to the boundaries of the environment [1,2]. However, further studies indicated that the geometry of the environment can influence the hexagonal grid cell pattern even in familiar environments [3]. Perhaps, the most extreme example of this effect is the pattern of grid cell firing on a linear track [4].

It has been suggested that grid cells provide a ‘metric system’ for navigation because their periodic pattern is invariant to animal's speed and behaviour during exploration [1,5]. Notably, hexagonal symmetry is not a fundamental requirement for metric representation of the environment. For instance, a pattern with 90° symmetry could equally well form the basis for a metric system (a grid). Indeed, before grid cells were discovered, a square lattice was used in earlier robot navigational models to represent the metric system for place cell generation [6,7].

How is the grid cell firing pattern generated? On the one hand, it could be driven predominantly by self-motion cues whereby an animal tracked the distance it moved in a particular direction (e.g. by step counting) and summed these distances across time [5,8–13]. Such path-integration-based navigation is inherently susceptible to idiothetic noise and the accumulation of errors. Hence, it has been widely suggested that the accumulating error can be corrected with a cue-driven feedback coming from place cells and/or boundary vector cells [5,8–14]. In addition, sensory-driven feedback might also serve as ‘an anchor’ that fixes grid offset resulting in a reproducible grid cell pattern during multiple visits to the same environment. It should be noted that in this type of model, grid cells are generated primarily from self-motion cues and sensory-driven information serves exclusively as a supporting signal to stabilize the grid cell pattern.

Alternatively, it has been suggested that the grid pattern may be generated primarily from sensory-cue-driven inputs [15]. In this case, place-like representations may arise from external visual cues [16–18] and other sensory modalities (especially when the visual cues are not available) and serve as a primary input to drive the grid response [15].

Here, we further reconsider what main factors may contribute to grid cell pattern formation and how these factors interact to create the final grid cell response. In our model, we put forward the idea that a grid cell pattern emerges from the interaction between inputs related to the boundary of the environment (which essentially defines its geometry) and place-like inputs which can either derive primarily from sensory-driven cell responses (as in [15]) or could also be generated mainly using self-motion cues (as in [19]) or potentially have access to both. The idea is inspired by the experimental observation that grid cells can have non-hexagonal symmetrical properties and that their symmetry can be strongly influenced by the geometry of the environments [20]. We introduce a mathematical model which describes the interaction between fields and boundaries to study how differently shaped environments affect the grid cell pattern. We also present some experimental evidence demonstrating the validity and the power of this model.

## 2. Material and methods

Eight male Lister hooded adult rats were used for the experiments. Under surgical anaesthesia, they were chronically implanted in the left hemisphere with a microdrive (Axona Ltd) loaded with four tetrodes. Tetrodes were aimed at the superficial layers of the medial part of dorso-caudal mEC and adjacent PaS (4.3–4.4 mm lateral to the midline; 0.3–0.4 mm anterior to the sinus and at an angle between 0° and 4° to the front plane; 1.5 mm below the pia).

Recordings took place in a familiar square (1.3 × 1.3 m (seven rats) or 0.9 × 0.9 m (one rat)) enclosure while the animals foraged for sweetened rice.

## 3. Field–boundary interaction model

**Figure 1.**
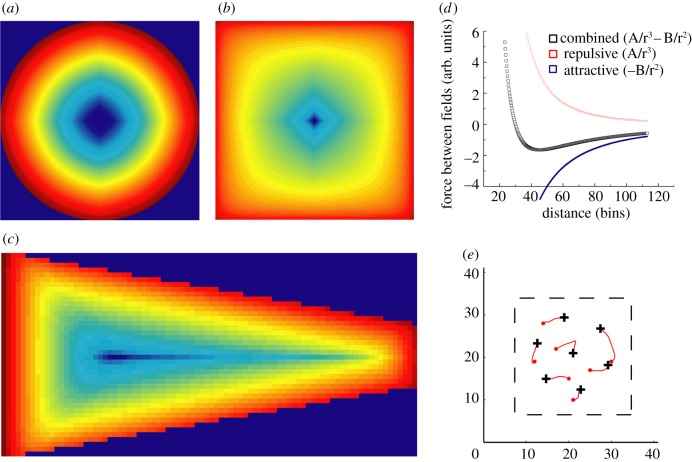
Boundary and field force profiles. Typical examples of boundary force profiles in (*a*) a circular, (*b*) a square and (*c*) a trapezoidal environment. The forces are shown on a logarithmic scale. (*d*) A typical example of a field-to-field interaction force along a single direction (see main text for equation (2.1)). (*e*) Fields converging to stable positions governed by boundary-to-field and field-to-field interaction forces. Red dots represent randomly assigned starting positions of the fields, red curving lines correspond to their convergence trajectories and crosses represent their stable final positions. Axes are shown in 2.5 cm bins. (Online version in colour.)

In this model, we describe the firing patterns of classical hexagonal grid cells and other spatially periodic non-grid cells as an asymptotic state of interacting fields with Gaussian-tuned responses. First, we assume an abstract semi-infinite two-dimensional plane with *N* number of fields. Each field interacts with the other field via ‘force’ *U*_field_ described by a modified Lennard–Jones formula (which was originally proposed by John Lennard–Jones to describe the interaction between a pair of neutral atoms or molecules; figure 1*d*)2.1
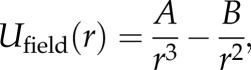
where *r* is a distance between field centres; *A* and *B* are fixed parameters related to the optimal spacing between the fields which defines its scale. The attractive and repulsive interaction forces are balanced (i.e. a total force reaches zero) when the fields are at *r*_scale_ distance from each other2.2
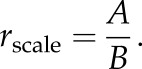
The main idea of our model is that place field inputs compete with each other by simultaneously attracting each other but also trying to suppress one another when they are too close. The suppression is mediated by the network of inhibitory neurons. The interaction is best approximated when in addition to repulsive force (described by *A*/*r*^3^ in equation (2.1)) there is an attractive force (described by *B*/*r*^2^ in equation (2.1)) which keeps the place-like firing at an optimal distance from each other.

In the simulations, we initially distribute the centres of the fields randomly across the environment. Given the interaction described in equation (2.1), they should depart from their initial positions, and finally reach the equilibrium state (figure 1*e*) at *r*_scale_ distance from each other. The resulting stable pattern would exhibit a hexagonal symmetry appearing as a grid cell pattern with a scale defined by *r*_scale_. The number of fields is defined by cell-intrinsic properties (such as its integration time constant or intrinsic oscillation frequency [21]).

Can we capture cases where spatially periodic cells show a periodic band-like pattern? Indeed, if the force *U*_field_ is equal across all the directions then it would be impossible to result in periodic band-like firing as observed in [20]. To account for that, an additional directional input is required. Equation (2.1) should be modified2.3
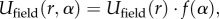
where *f*(**α**) is a direction-dependent function similar to a head direction response function or a combination of such functions.

Now let us suppose that the semi-infinite abstract space is perturbed by introducing some boundaries (e.g. a circle, a square, etc.) which affect each field with the exponentially decreasing ‘force’ *U*_boundary_ described as2.4
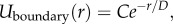
where *C* and *D* are fixed parameters representing the strength and the profile of this interaction. The repulsive boundary force field *U*_boundary_ is generated at every environment point by all the boundaries and is directed perpendicularly to the corresponding boundary (figure 1*a–c*). Depending on the parameters *A*, *B*, *C* and *D*, the fields’ distribution within the boundaries can exhibit the whole range of possible symmetrical properties. Thus, we can experimentally probe the same cell in different shape environments (e.g. a circle, a square, etc.), estimate these parameters and in principle be able to predict how this pattern may be transformed in other shaped enclosures (e.g. a trapezoid, a linear track, etc.) assuming the values of the *A*, *B*, *C* and *D* parameters remain constant.

**Figure 2.**
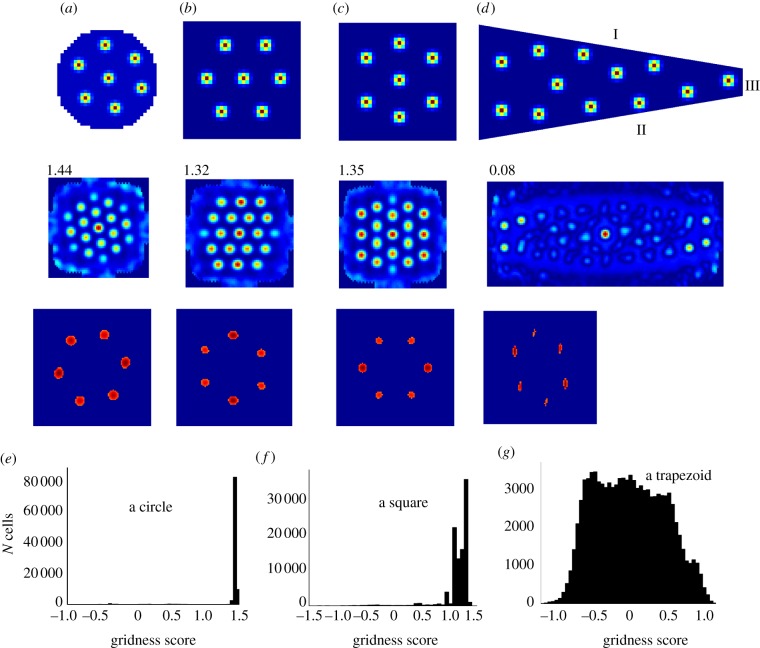
Simulated grid cell firing patterns in differently shaped environments. Typical examples of simulated grid cell firing patterns in (*a*) circular, (*b*,*c*) square and (*d*) trapezoidal enclosures. Firing-rate maps (top row), spatial autocorrelograms (second row) with a gridness score specified on the top left corner, and two-dimensional Fourier spectrograms (bottom row). Two distinct grid cell populations are generated in a square environment: one aligned to a horizontal wall (*b*) and another aligned to a vertical wall (*c*). The overall distribution of simulated gridness scores in a circle (*e*), square (*f*) and trapezoid (*g*). All grid firing patterns were generated using the same field–boundary interaction parameters. (Online version in colour.)

We have simulated patterns in circular, square and trapezoidal environments using the same parameter set (100 000 simulations for each shape). Interestingly, while equilibrium state mostly exhibits hexagonal symmetry in square and circular environments (figure 2*a–c*,*e*,*f*), the symmetry significantly departs from hexagonal in a trapezoidal enclosure (figure 2*d*,*g*).

**Figure 3.**
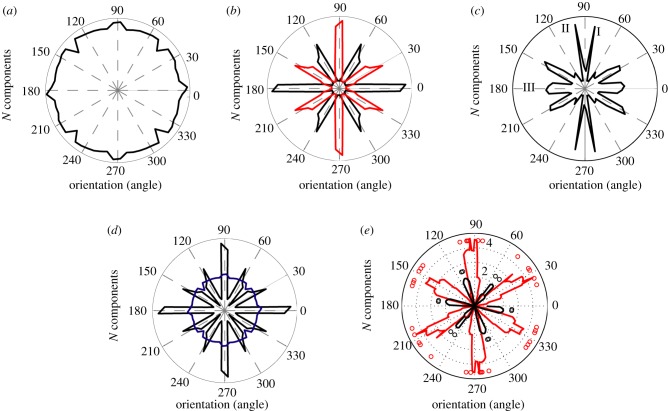
Orientations of the main Fourier components of simulated grid cells firing in (*a*) a circle, (*b*) square and (*c*) trapezoid. In (*b*), red and black lines represent orientations aligned to the horizontal (0°) and vertical (90°) walls, respectively. (*d*) Orientations in circular (blue) and square (black) environments superimposed on each other clearly demonstrate the significant clustering of grid cell orientations in a square but not a circle. (*e*) The overall distribution of means of the main Fourier components of all grid cells in a square (recordings done in eight rats, with nine different grid cell modules). Red and black dots represent main mean orientations from each grid module. Note the two different orientations of the grids in the same square. (Online version in colour.)

Another important observation was revealed by these simulations: namely, one of the main Fourier components should tend to align to one of the walls. As a result, in a circle environment the grid pattern may show any preferred orientation (figure 3*a*,*d*). However, in a square environment one of the main Fourier components should tend to cluster around 0° or 90° orientation (aligned to a vertical or a horizontal wall, respectively). In this case, if different grid cell modules act independently [22], there should exist two different grid cell populations rotated by 30° (figures 2*b*,*c* and 3*b*,*d*).

A trapezoidal environment presents a more complex case (100 000 simulations). In our simulations, we used a trapezoid with dimensions of 190 cm × 20 cm × 80 cm (as in figure 1*c*). Five main Fourier components were observed (figures 2*d* and 3*c*): two aligned to each of the longer walls (I and II), two other components rotated by 60° and 120° with respect to the first one and one additional component aligned to the shorter walls (III at 180°). The components aligned to the longer walls (I and II, figures 2*d* and 3*c*) are separated by 12° which is also the angle between the orientations of the longer walls. Interestingly, the component aligned to the shorter walls did not evoke the generation of two more components rotated by 60° and 120° from it. Hence, it suggests that by changing the ratio between shorter and longer walls, we should be able to change the weights of main Fourier components and even observe new ones oriented by 30° or 60° to the existing ones.

## 4. Effects of environment geometry on grid cell pattern: experimental observations versus theoretical predictions

It has been suggested that grid cells represent a subset of a more general group of spatially periodic cells which together comprise the majority of cells in the superficial layers of mEC and parasubiculum (PaS) [20]. In circular and rectangular enclosures, hexagonal symmetry was shown to be the most stable firing pattern, hence many cells in mEC represent the classic grid cells (75/156 or 48%, *N*_rats_ = 5). However, occasionally spatially periodic cells undergo a pattern transformation whereby they lose or gain hexagonal symmetry. Spontaneous transformation (i.e. when no experimental condition is changed) is quite rare (approx. 11% in a 1.3 × 1.3 m^2^ square environment [20]). On the other hand, the transformation probability becomes significantly higher between two geometrically different enclosures (approx. 32%).

We also investigated whether orientations of the main Fourier components tend to align along the walls of a square environment as predicted by the field–boundary interaction model. Figure 3*e* shows that one of the main grid orientations (out of three) is always aligned to one of the walls (*N*_rats_ = 8; *N*_GCmodules_ = 9). In one case where we simultaneously recorded two different grid cell modules (with different scales and main orientations rotated by 30° from each other), both modules were aligned along different walls of the environment [20].

## 5. Model predictions

Although at this stage the field–boundary interaction model has a purely descriptive nature, it can already explain some existing experimental observations and make explicit predictions with regard to some untested grid cell response properties:

(1) The model predicts that the geometry of the environment should have a strong effect on the symmetrical properties of the grid cell firing. For instance, in a quite irregular environment, for example a trapezoid, grid cells should often lose their hexagonal symmetry and gain main components aligned to one of the walls or aligned at multiples of 60° to the walls. The length of the wall can affect the strength of its related components.
(2) The immediate consequence of the first prediction is that in a square environment two different grid cell populations rotated by approximately 30° should exist aligned to horizontal and vertical walls. In a novel square or circular environment, grid cells should initially emerge as less regular and over time converge to hexagonally symmetrical patterns as the animal gains experience in this environment.
(3) In novel environments of identical geometry, grid cells should have different offsets. This follows from the fact that the offset depends on the initial condition and these will differ in novel environment but not in familiar environments.
(4) Because of the inhibitory ‘force’ coming from the boundary, we predict some inhibitory neurons firing only at the border (inhibitory border cells) and projecting to CA1 place cells. These projections could come either from mEC [23] or from subiculum [24].
(5) Grid cells with different scales should respond differently to the geometry of the environment. This is owing to the fact that the shape of the force-tuning curve (equation (2.1); figure 1*d*) will vary with different grid scales.
(6) The insertion of the boundary into a square environment should result in the shift and rearrangement of grid fields away from the inserted boundary. The degree of rearrangement should depend on the ratio between the length of the inserted boundary and the grid scale. Grid cells with very small scale compared with the boundary size should be perturbed mostly in the vicinity of the boundary, whereas grid cells with scales much larger than the boundary size should not be affected very much. The strongest effect should be observed on grid cells with scales close to the size of the boundary.

## 6. A possible physiological implementation

**Figure 4.**
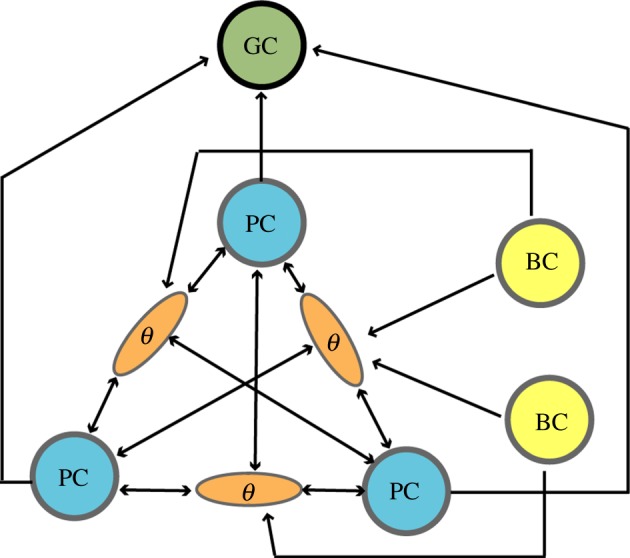
A schematic of possible underlying mechanism of the force–boundary interaction model. Place cells are shown in blue, a spatially periodic cell including grid cell in green, border cells in yellow and theta cells are shown in brown. Place cells (blue) and border cells (yellow) are interconnected via inhibitory theta interneuron network (brown). The ‘winner’ place cells drive the firing pattern of the afferent grid cell (green). (Online version in colour.)

A possible physiological mechanism underlying the field–boundary interaction model is outlined in figure 4. Here, the field-like inputs are received from competing place cell networks which are all connected to each other via inhibitory interneurons. Afferent place cells with adjacent fields compete with each other till only the ones firing at distance approximately *r*_scale_ remain active. Additionally, boundary cells from subiculum or mEC [25,26] project to the local place cell inhibitory network selectively inhibiting as a function of distance to the boundaries. Alternatively, a new cell type, inhibitory boundary cells, could send projections to place cells. Indirect observations suggesting the existence of the inhibitory boundary cells come from the recordings of ‘boundary-off cells’ [24]. If our model is correct, it suggests that place cells form a primary substrate of spatial representation generated by sensory cues (as in [15]) and/or self-motion cues (as in [19]). We predict that CA1 and subiculum cells send direct inputs to the deep layers of mEC and PaS where grid cells start emerging. These grid cells then have direct projections to the superficial layers of mEC where the grid cells are also found. Importantly, deep layers of mEC send prominent inputs to other neocortical areas. These afferents may serve as a substrate for combining all the sensory information in a single spatial framework.

It still must be addressed how hippocampal place cells receive their sensory and self-motion-driven inputs because their strongest afferents are arriving from medial and lateral EC [27]. Place cells can be generated in the absence of grid cells; therefore, at least the sensory information must reach place cells even without regular grid cell activity. This observation suggests that grid cells may serve as a relay of spatial information from the hippocampus.

## 7. Conclusion

We have introduced a field–boundary interaction model attempting to explain recent observations that the geometry of the environment can have a profound effect on the grid cell firing pattern beyond a simple stretching or rescaling of the grid [3,20]. In this model, the grid cell pattern emerges from the interaction between competing place-like inputs and boundary inputs. At this stage, it must be stressed that our model is purely descriptive. However, it allows us to make clear experimentally testable predictions about the firing properties of grid cells under different experimental conditions (e.g. different shape enclosures, responses to barriers inside the enclosures, etc.), which if confirmed can give invaluable insights about what the main factors of grid cell formation are, how they interact and what functional role grid cells may have.

The current dominant theory is that different grid cells linearly sum up to generate a place cell response [1,2,8,9,11,12,28–31]. However, despite the intuitive appeal of this view, there is actually little data currently available to support it. Several recent experimental studies have shown that place cells can form and exist even in the absence of grid cell inputs [32–34]. Interestingly, there are data to support the opposite view, that grid cells are formed from place cells, as grid cells are not present without the input from hippocampal cells [35]. Moreover, in novel environments both place cells and grid cells undergo remapping and field expansion but place cells expand by smaller amounts and converge to their sharply state more rapidly compared with grid cells [36]. Taken together, these results suggest that the widespread assumption that grid cells are the only input to place cells and linearly sum up to generate them should be reconsidered. The existence of place cells without grid cells and their different response dynamics to novelty (place cells fine tune faster) of course cannot serve as a proof that grid cells are formed from place cells, and to date there is no obvious experiment that could easily address this question. The observations from our model offer such an experiment by making clear testable predictions.

## Notes

All animal procedures were carried out in accordance with the UK Animals (Scientific Procedures) Act 1986.

## References

[1] Hafting T Fyhn M Molden S Moser M-B Moser EI 2005 Microstructure of a spatial map in the entorhinal cortex. Nature 436, 801–806. (10.1038/nature03721) 15965463

[2] Fyhn M Hafting T Treves A Moser M-B Moser EI 2007 Hippocampal remapping and grid realignment in entorhinal cortex. Nature 446, 190–194. (10.1038/nature05601) 17322902

[3] Barry C Hayman R Burgess N Jeffery KJ 2007 Experience-dependent rescaling of entorhinal grids. Nat. Neurosci. 10, 682–684. (10.1038/nn1905) 17486102

[4] Brun VH Solstad T Kjelstrup KB Fyhn M Witter MP Moser EI Moser M-B 2008 Progressive increase in grid scale from dorsal to ventral medial entorhinal cortex. Hippocampus 18, 1200–1212. (10.1002/hipo.20504) 19021257

[5] McNaughton BL Battaglia FP Jensen O Moser EI Moser M-B 2006 Path integration and the neural basis of the ‘cognitive map’. Nat. Rev. Neurosci. 7, 663–678. (10.1038/nrn1932) 16858394

[6] Elfes A 1989 Using occupancy grids for mobile robot perception and navigation. IEEE J. Robot. Autom. 22, 46–57.

[7] Moravec HP 1988 Sensor fusion in certainty grids for mobile robots. AI Mag. 9, 61–74.

[8] Burgess N Barry C O'Keefe J 2007 An oscillatory interference model of grid cell firing. Hippocampus 17, 801–812. (10.1002/hipo.20327) 17598147 PMC2678278

[9] Hasselmo ME 2008 Grid cell mechanisms and function: contributions of entorhinal persistent spiking and phase resetting. Hippocampus 18, 1213–1229. (10.1002/hipo.20512) 19021258 PMC2614862

[10] Fuhs MC Touretzky DS 2006 A spin glass model of path integration in rat medial entorhinal cortex. J. Neurosci. 26, 4266–4276. (10.1523/JNEUROSCI.4353-05.2006) 16624947 PMC6674007

[11] Fiete IR Burak Y Brookings T 2008 What grid cells convey about rat location. J. Neurosci. 28, 6858–6871. (10.1523/JNEUROSCI.5684-07.2008) 18596161 PMC6670990

[12] Burak Y Fiete IR 2009 Accurate path integration in continuous attractor network models of grid cells. PLoS Comput. Biol. 5, e1000291. 19229307 10.1371/journal.pcbi.1000291 PMC2632741

[13] Blair HT Gupta K Zhang K 2008 Conversion of a phase- to a rate-coded position signal by a three-stage model of theta cells, grid cells, and place cells. Hippocampus 18, 1239–1255. (10.1002/hipo.20509) 19021259 PMC2814603

[14] Monaco JD Knierim JJ Zhang K 2011 Sensory feedback, error correction, and remapping in a multiple oscillator model of place-cell activity. Front. Comput. Neurosci. 5, 00039 (10.3389/fncom.2011.00039) PMC3182374 21994494

[15] Kropff E Treves A 2008 The emergence of grid cells: intelligent design or just adaptation? Hippocampus 18, 1256–1269. (10.1002/hipo.20520) 19021261

[16] Wiskott L 2003 Slow feature analysis: a theoretical analysis of optimal free responses. Neural Comput. 15, 2147–2177. (10.1162/089976603322297331) 12959670

[17] Franzius M Sprekeler H Wiskott L 2007 Slowness and sparseness lead to place, head-direction, and spatial-view cells. PLoS Comput. Biol. 3, e166 (10.1371/journal.pcbi.0030166) 17784780 PMC1963505

[18] Barry C 2006 The boundary vector cell model of place cell firing and spatial memory. Rev Neurosci 17, 71–97. (10.1515/REVNEURO.2006.17.1-2.71) 16703944 PMC2677716

[19] Samsonovich A McNaughton BL 1997 Path integration and cognitive mapping in a continuous attractor neural network model. J. Neurosci. 17, 5900–5920. 9221787 10.1523/JNEUROSCI.17-15-05900.1997 PMC6573219

[20] Krupic J Burgess N O'Keefe J 2012 Neural representations of location composed of spatially periodic bands. Science 337, 853–857. (10.1126/science.1222403) 22904012 PMC4576732

[21] Giocomo LM Zilli EA Fransén E Hasselmo ME 2007 Temporal frequency of subthreshold oscillations scales with entorhinal grid cell field spacing. Science 315, 1719–1722. (10.1126/science.1139207) 17379810 PMC2950607

[22] Stensola H Stensola T Solstad T Fraland K Moser M-B Moser EI 2012 The entorhinal grid map is discretized. Nature 492, 72–78. (10.1038/nature11649) 23222610

[23] Melzer S Michael M Caputi A Eliava M Fuchs EC Whittington MA Monyer H 2012 Long-range-projecting GABAergic neurons modulate inhibition in hippocampus and entorhinal cortex. Science 335, 1506–1510. (10.1126/science.1217139) 22442486

[24] Stewart S Jeewajee A Wills TJ Burgess N Lever C 2014 Boundary coding in the rat subiculum. Phil. Trans. R. Soc. B 369, 20120514 (10.1098/rstb.2012.0514) 24366128 PMC3866438

[25] Solstad T Boccara CN Kropff E Moser M-B Moser EI 2008 Representation of geometric borders in the entorhinal cortex. Science 322, 1865–1868. (10.1126/science.1166466) 19095945

[26] Lever C Burton S Jeewajee A O'Keefe J Burgess N 2009 Boundary vector cells in the subiculum of the hippocampal formation. J. Neurosci. 29, 9771–9777. (10.1523/JNEUROSCI.1319-09.2009) 19657030 PMC2736390

[27] van Strien NM Cappaert NLM Witter MP 2009 The anatomy of memory: an interactive overview of the parahippocampal–hippocampal network. Nat. Rev. Neurosci. 10, 272–282. (10.1038/nrn2614) 19300446

[28] Solstad T Moser EI Einevoll GT 2006 From grid cells to place cells: a mathematical model. Hippocampus 16, 1026–1031. (10.1002/hipo.20244) 17094145

[29] Burgess N 2008 Grid cells and theta as oscillatory interference: theory and predictions. Hippocampus 18, 1157–1174. (10.1002/hipo.20518) 19021256 PMC3196519

[30] Moser EI Kropff E Moser M-B 2008 Place cells, grid cells, and the brain's spatial representation system. Annu. Rev. Neurosci. 31, 69–89. (10.1146/annurev.neuro.31.061307.090723) 18284371

[31] Brun VH Leutgeb S Wu H-Q Schwarcz R Witter MP Moser EI Moser M-B 2008 Impaired spatial representation in CA1 after lesion of direct input from entorhinal cortex. Neuron 57, 290–302. (10.1016/j.neuron.2007.11.034) 18215625

[32] Wills TJ Cacucci F Burgess N O'Keefe J 2010 Development of the hippocampal cognitive map in preweanling rats. Science 328, 1573–1576. (10.1126/science.1188224) 20558720 PMC3543985

[33] Langston RF Ainge JA Couey JJ Canto CB Bjerknes TL Witter MP Moser EI Moser M-B 2010 Development of the spatial representation system in the rat. Science 328, 1576–1580. (10.1126/science.1188210) 20558721

[34] Koenig J Linder AN Leutgeb JK Leutgeb S 2011 The spatial periodicity of grid cells is not sustained during reduced theta oscillations. Science 332, 592–595. (10.1126/science.1201685) 21527713

[35] Bonnevie T Dunn B Fyhn M Hafting T Derdikman D Kubie JL Roudi Y Moser EI Moser M-B 2013 Grid cells require excitatory drive from the hippocampus. Nat. Neurosci. 16, 309–317. (10.1038/nn.3311) 23334581

[36] Barry C Ginzberg LL O'Keefe J Burgess N 2012 Grid cell firing patterns signal environmental novelty by expansion. Proc. Natl Acad. Sci. USA 109, 17 687–17 692. (10.1073/pnas.1209918109) PMC3491492 23045662

